# Genetic parameters and genome-wide association study of digital cushion thickness in Holstein cows

**DOI:** 10.3168/jds.2022-22035

**Published:** 2022-10

**Authors:** Matthew Barden, Bingjie Li, Bethany E. Griffiths, Alkiviadis Anagnostopoulos, Cherry Bedford, Androniki Psifidi, Georgios Banos, Georgios Oikonomou

**Affiliations:** 1 Department of Livestock and One Health, Institute of Infection, Veterinary and Ecological Sciences, University of Liverpool, Leahurst Campus, Liverpool, CH64 7TE, United Kingdom; 2 Animal & Veterinary Sciences, SRUC, Roslin Institute Building, Easter Bush, Midlothian, EH25 9RG, United Kingdom; 3 Department of Clinical Science and Services, Royal Veterinary College, North Mymms, Hertfordshire, AL9 7TA, United Kingdom

**Keywords:** lameness, dairy cattle, digital cushion, genome-wide association study

## Abstract

The digital cushion is linked to the development of claw horn lesions (CHL) in dairy cattle. The objectives of this study were to (1) estimate genetic parameters for digital cushion thickness (DCT), (2) estimate the genetic correlation between DCT and CHL, and (3) identify candidate genes associated with DCT. A cohort of 2,352 Holstein dairy cows were prospectively enrolled on 4 farms and assessed at 4 time points: before calving, immediately after calving, in early lactation, and in late lactation. At each time point, CHL was recorded by veterinary surgeons, and ultrasonographic images of the digital cushion were stored and retrospectively measured at 2 anatomical locations. Animals were genotyped and pedigree details extracted from the national database. Genetic parameters were estimated following a single-step approach implemented in AIREMLF90. Four traits were analyzed: the 2 DCT measurements, sole lesions (sole hemorrhage and sole ulcers), and white line lesions. All traits were analyzed with univariate linear mixed models; bivariate models were fit to estimate the genetic correlation between traits within and between time points. Single-marker and window-based genome-wide association analyses of DCT traits were conducted at each time point; candidate genes were mapped near (<0.2 Mb) or within the genomic markers or windows with the largest effects. Heritability estimates of DCT ranged from 0.14 to 0.44 depending on the location of DCT measurement and assessment time point. The genetic correlation between DCT and sole lesions was generally negative, notably between DCT immediately after calving and sole lesions in early or late lactation, and between DCT in early or late lactation and sole lesion severity in early or late lactation. Digital cushion thickness was not genetically correlated with white line lesions. A polygenic background to DCT was found; genes associated with inflammation, fat metabolism, and bone development were mapped near or within the top markers and windows. The moderate heritability of DCT provides an opportunity to use selective breeding to change DCT in a population. The negative genetic correlation between DCT and sole lesions at different stages of production lends support to current hypotheses of sole lesion pathogenesis. Highlighted candidate genes provide information regarding the complex genetic background of DCT in Holstein cows, but further studies are needed to explore and corroborate these findings.

## INTRODUCTION

The digital cushion of the bovine claw is composed of 3 parallel pads of soft fat and loose connective tissue that are thought to play an important role in protecting the corium from mechanical forces in the foot (Lischer and Ossent, 2002; Räber et al., 2004; Räber et al., 2006). New horn develops via cornification of keratinocytes, cells that are nutritionally dependent on the corium (Hirschberg et al., 2001), and damage to the corium consequently leads to the production of poor quality horn (Hoblet and Weiss, 2001). The term “claw horn (disruption) lesion” (**CHL**) refers to foot lesions that arise from damaged corium, including sole hemorrhage (**SH**), sole ulcers (**SU**), and white line lesions (**WL**).

In 1999, Kofler et al. (1999) described an ultrasonographic approach to visualize the digital cushion in live animals. The digital cushion thickness (**DCT**) has since been measured in a succession of studies, with a general agreement that a thin DCT represents an increased risk of subsequent CHL development (Machado et al., 2011; Toholj et al., 2014; Newsome et al., 2017a; Stambuk et al., 2018; Griffiths et al., 2020). To date, studies that have focused on CHL as individual lesions have only demonstrated an association between DCT and SH or SU (Newsome et al., 2017a; Griffiths et al., 2020). Therefore, the contribution of WL to the previously reported associations between DCT and CHL development is unclear.

Variation in DCT has been summarized as being conditional on a combination of genetic, developmental, and pathological factors (Wilson et al., 2021); the genetic background of DCT is the focus of our study. The heritability of DCT, measured using ultrasonography, has been estimated to be 0.33 (±0.09) using pedigree relationships (Oikonomou et al., 2014) and 0.31 (±0.13) from multiple genomic analyses (Stambuk et al., 2020b). One other study, which prospectively enrolled cows specifically for research purposes, reported the heritability of DCT in recently calved cows to be 0.23 (±0.12) (Sánchez-Molano et al., 2019). A negative genetic correlation has been reported between DCT and CHL incidence (Oikonomou et al., 2014); however, the separate genetic relationships between DCT and sole lesions (SH and SU) and between DCT and WL are unknown.

The thickness of the digital cushion has been observed to change around calving and throughout lactation, but with inconsistencies regarding the exact changes that occur (Newsome et al., 2017b; Stambuk et al., 2018; Griffiths et al., 2020; Bach et al., 2021). In some cases, the DCT has been reported to be thinnest during early lactation, which has been suggested to be a consequence of fat tissue mobilization and a reflection of the concurrent nadir in body condition (Bicalho et al., 2009; Griffiths et al., 2020). Alternatively, other studies have observed the thinnest DCT immediately after calving (Newsome et al., 2017b; Bach et al., 2021), which is thought to be the result of compression due to periparturient laxity in the suspensory apparatus of the distal phalanx (Tarlton et al., 2002; Knott et al., 2007). Therefore, it would be useful to estimate the genetic parameters of DCT at different periods during lactation, particularly as stage of lactation has been shown to affect genetic parameter estimates for other foot lesion traits (Gernand et al., 2013).

Genome-wide association (**GWA**) studies have used genomic markers to map QTL that may be associated with the genetic variance of DCT. Two recent GWA studies of around 600 Holstein and Jersey cows characterized DCT as a complex trait and identified candidate genes related to inflammation, fat tissue deposition, bone growth, and keratinocyte function (Stambuk et al., 2020a,b). Further studies are needed to corroborate previously highlighted QTL as well as to explore QTL for DCT in different populations.

The objectives of our study were to (1) estimate genetic parameters for DCT at different stages during a production cycle; (2) estimate the genetic correlation between DCT and CHL, evaluating sole lesions (SH and SU) and WL separately; and (3) identify QTL associated with DCT.

## MATERIALS AND METHODS

### Study Design and Population

The study was conducted following ethical approval by the University of Liverpool Research Ethics Committee (VREC269a, VREC466ab) and procedures regulated by the Animals (Scientific Procedures) Act were conducted under a United Kingdom (UK) Home Office License (P191F589B).

A prospective cohort study was designed to measure the DCT in dairy cows and record CHL at 4 time points during a production cycle. Data were collected from 4 dairy herds (A–D) in the northwest of the United Kingdom. These herds were selected for convenience based on the practicalities of frequent visits and assessments. Herds A to C housed lactating cows year round, milked cows 3 times daily, and recorded 305-d milk yields of approximately 11,000 to 11,500 L. Herd D housed lactating cows year round except for lower yielding cows, which were grazed during the summer; cows were milked twice daily, and the 305-d milk yield was approximately 9,000 L. Parous cows in all herds were routinely foot-trimmed twice a year before drying-off and at 60 to 120 d after calving. In all herds, lactating cows were regularly footbathed after milking. Herd A footbathed cows 3 times a week with either copper sulfate or formalin, herd B footbathed cows twice daily with formalin, herd C footbathed cows daily with either copper sulfate or formalin, and herd D footbathed cows 3 times a week with formalin.

All animals that were registered as Holsteins and expected to calve between April and December 2019 were prospectively enrolled with no additional inclusion or exclusion criteria applied. A total of 2,352 animals were enrolled. Data were collected during weekly or twice weekly visits to each herd from February 2019 to July 2020 (with a break from March to June 2020 due to COVID-19 restrictions). Animals were assessed for foot lesions at 4 time points: before parturition (**T1-Precalving**), immediately after parturition (**T2-Calving**), in early lactation around the time of peak milk yield (**T3-Early**), and in late lactation (**T4-Late**). Sample size was determined by resource constraints; all eligible animals were enrolled until the final assessments (T4-Late) began, at which point further enrollments ended because simultaneous data collection at 4 time points was not feasible.

### Data Collection

At each assessment time point, animals were restrained in a foot-trimming crush and, if foot-trimming had not been conducted during the visit, the claw horn on the sole of each foot was lightly trimmed to allow inspection of foot lesions. On each claw, CHL were recorded using case definitions as described in the International Committee for Animal Recording claw health atlas (Egger-Danner et al., 2020). All CHL were graded according to severity (Table 1), broadly comparable to absent (score 0), mild (score 1), moderate (score 2), and severe (score 3). All foot lesions were examined and recorded by qualified veterinary surgeons; over 90% were assessed by a single researcher, and the remainder by 3 other researchers.

**Table 1:** Case definitions and severity grading system for sole hemorrhage, sole ulcers, and white line lesions

Lesion	Case definition	Severity grading
Sole hemorrhage	Discoloration of the sole horn	Grade 1: light pink lesion <2 cm diameter or diffuse discoloration of sole Grade 2: light pink lesion ≥2 cm diameter or dark pink/purple lesion <2 cm diameter Grade 3: dark pink/purple lesion ≥2 cm diameter or discoloration with blue tinge
Sole ulcer	Exposure of fresh or necrotic corium	Grade 1: <2 cm diameter lesion covered by thin layer of horn before modeling Grade 2: ≥2 cm diameter lesion with <1.5 cm granulation tissue protruding through horn Grade 3: ≥1.5 cm granulation tissue protruding through horn or secondary bacterial infection
White line lesion	Lesion localized to the white line region	Grade 1: hemorrhage of the white line or discoloration or separation of the white line which disappears after limited trimming Grade 2: deeper separation or discoloration of the white, lesion is still present after limited trimming Grade 3: separation of the white line which extends to the corium, purulent exudate or necrotic tissue may be present

After CHL had been recorded, the digital cushion was imaged in the lateral claw of the left-hind digit using B-mode ultrasonography with the foot still lifted off the ground (i.e., non-weight-bearing), as previously described (Kofler et al., 1999). This site was chosen because the lateral claw of the hindlimb digit is the most common site of CHL development (Murray et al., 1996). Time constraints did not allow us to scan more than one claw per cow, and the left-hind digit was arbitrarily chosen over the right-hind digit. A 5-cm linear probe inside a gel standoff was used with a DRAMIŃSKI Vet 4 Mini ultrasound machine (DRAMIŃSKI S. A.); frequency was set to 6 MHz and image depth to 4 cm. The probe was placed on the midline of the sole, and the ultrasound image was stored when the digital cushion, arch of the distal phalanx, and flexor tuberosity of the distal phalanx were visible.

The data collection procedure was the same at all time points with 2 exceptions. At T2-Calving on herd C, only hind feet were assessed to reduce the handling time of cows that had recently calved; this approach was only required in this herd owing to the large numbers of cows calving each week. The other exception was at the T4-Late assessments, which resumed in June 2020 following a break due to COVID-19 restrictions; data collection from this point onwards was more limited owing to social distancing protocols, and digital cushion images were not collected.

Following completion of data collection, DCT was measured on stored images by a single researcher using ImageJ (Schneider et al., 2012). Images were first relabeled so the researcher was blinded to details regarding image collection such as time point, farm, or concurrent lesions. Two measurements were recorded, in both cases between the internal aspect of the sole horn and the distal aspect of the distal phalanx. The first measurement was at the most proximal point in the arch of distal phalanx, representing the greatest thickness of the digital cushion (**DCT-MAX**); this point corresponds to the interconnecting abaxial and axial pads of the digital cushion (primarily the axial pad) in the midline of the claw (Räber et al., 2004). The second measurement was immediately distal to the flexor tuberosity of the distal phalanx (**DCT-FT**); at this point, the specific part of the digital cushion measured was assumed to be the middle pad in a typical cow (Räber et al., 2004). [For further details, readers are referred to Figure 1 in Newsome et al., (2017b), which shows a midline sagittal section of the bovine digit with corresponding ultrasound image; in this figure, site 2 corresponds to DCT-MAX and site 3 to DCT-FT.] We only recorded measurements if landmarks were clearly identifiable on each image; necessary landmarks included the interfaces between sole horn and soft tissue and the interface between soft tissue and distal phalanx. Additionally, the DCT-FT measurement was only taken if the point of the flexor tuberosity of the distal phalanx could be clearly identified.

Importantly, these ultrasonographic DCT measurements do not exclusively relate to the digital cushion and include all soft tissues between the sole horn and distal phalanx, which include connective tissue and the corium (Kofler et al., 1999; Räber et al., 2004). For this reason, DCT measured using ultrasonography is sometimes, more correctly referred to as sole soft tissue thickness (Newsome et al., 2017b; Griffiths et al., 2020); however, for consistency with the majority of published research in this area, DCT is used throughout this article.

### Trait Definitions

The 2 DCT measurements (DCT-MAX and DCT-FT) were analyzed as continuous traits. As per our research objectives, sole lesions (SH and SU) were analyzed separately from WL. It is thought that SH and SU represent different stages of the same disease process (Offer et al., 2000; Lischer and Ossent, 2002). Therefore, at each time point, the severity grades of SH and SU on each claw were combined so that the severity ranged from 0 to 6, with grades 1 to 3 directly corresponding to SH severity, and grades 4 to 6 corresponding to SU severity. The maximum severity of sole lesions from the medial and lateral claw of each foot was taken and then averaged across all feet to create a variable called “sole lesion severity” (**SL-Severity**). This approach was intended to capture the severity and distribution of sole lesions, while minimizing the diluting effect of healthy claws in animals with sole lesions. We followed the same process for WL (calculating the average of the most severe WL from each foot) to create a variable called “white line lesion severity” (**WL-Severity**). Both SL-Severity and WL-Severity were analyzed as continuous traits.

### Pedigrees and Genotypes

Pedigree details for the study population were extracted from the national database of dairy cattle by tracing back 7 generations for each animal. Blood samples were collected from the coccygeal vein of each animal into EDTA vacutainers and used to genotype each animal with the Illumina BovineSNP50 BeadChip (Illumina Inc.). Genotypes were subsequently imputed to 80K SNP genotypes by Edinburgh Genetic Evaluation Services (EGENES) using an in-house procedure that has been developed for all national genomic evaluations of dairy cattle in the United Kingdom. Briefly, this imputation process uses the Illumina BovineSNP50 BeadChip and Illumina BovineHD BeadChip (Illumina Inc.), in addition to other commercial genotyping arrays, extra gene tests, and large-effect sequence variants. Following imputation, genotypes included 79,051 SNP spanning the entire genome. Chromosomal locations of the imputed 80K SNP panel were drawn from the latest assembly of the *Bos taurus* genome (ARS-UCD 1.2) (Rosen et al., 2020).

Imputed genotypes were available for 2,250 animals. Genotype quality control was implemented using PREGSF90 (Aguilar et al., 2014) within the BLUPF90 software suite (Misztal et al., 2018). Quality control included the removal of SNP with a call rate <0.90 (n = 10,977), SNP with a minor allele frequency <0.05 (n = 3,008), monomorphic SNP (n = 36), or SNP showing a strong deviation (>0.15) from Hardy-Weinberg equilibrium (n = 14) (Wiggans et al., 2009). Additionally, animals were removed if sample call rate was <0.90 (n = 63) or parent-progeny Mendelian conflicts were present (n = 20). Quality control procedures resulted in a final data set of 2,167 animals with genotypes of 65,211 SNP.

### Genetic Parameter Estimation

Before genetic analyses, fixed effects were evaluated via mixed effect linear regression of repeated observations of each trait, with animal as a random effect in the model. This analysis was conducted in R (R Core Team, 2021) with the *lmerTest* package (Kuznetsova et al., 2017). The following fixed effects were tested: herd, parity, season of calving, season of assessment, days since calving, and the researcher who recorded CHL. The importance of fixed effects was determined by finding the multivariable model with the lowest Akaike information criteria. Season of calving had similar effects on model fit as season of assessment. The effect of which researcher examined and recorded CHL increased Akaike information criteria, so this term was not included in subsequent genetic analyses.

Genetic parameters of each trait (DCT-MAX, DCT-FT, SL-Severity, WL-Severity) were estimated at each time point separately (T1-Precalving, T2-Calving, T3-Early, T4-Late) with single-trait linear mixed models, resulting in a total of 16 univariate models. Models were fit using the average information restricted maximum likelihood algorithm, implemented in AIREMLF90 (Misztal et al., 2018). The genetic parameters of each trait at each of the 4 time points were estimated with following univariate animal model:

[1] y=Xb+Za+e,

where **y** is a vector of one of the 4 traits (DCT-MAX, DCT-FT, SL-Severity, or WL-Severity); **b** is a vector of the fixed effects including herd (4 levels), parity (5 levels, 5th level = 5th parity and greater), season of calving (2 levels, April–September or October–March), and days relative to parturition (continuous variable); **a** is a vector of random additive genetic effects for each animal; **e** is vector of random residual effects; and **X** and **Z** are incidence matrices for **b** and **a**, respectively. Random effects were assumed to be normally distributed with a mean of zero and covariance structure of

[2] $$\mathrm{var}\left[\begin{array}{c} \text{a}\\ \text{e} \end{array}\right]=\left[\begin{array}{cc} \text{H}\sigma_{\text{a}}^{2} & \text{0}\\ \text{0} & \text{I}\sigma_{\text{e}}^{\text{2}} \end{array}\right]$$

where $\sigma_{\text{a}}^{2}$ is the additive genetic variance; $\sigma_{\text{e}}^{2}$ is the residual variance; **I** is an identity matrix and **H** is the relationship matrix incorporating pedigree and genomic information in a single-step genomic analyses framework (Legarra et al., 2009). The inverse of **H** (Aguilar et al., 2010; Christensen and Lund, 2010) is defined as follows:

[3] $${\text{H}}^{-1}={\text{A}}^{-1}+\left[\begin{array}{cc} 0 & 0\\ 0 & \left({\text{G}}^{-1}-{\text{A}}_{22}^{-1}\right) \end{array}\right],$$

where **A** is the pedigree relationship matrix; **G** is the genomic relationship matrix, and **A**_22_ is the pedigree relationship matrix for genotyped animals. The **A** matrix includes inbreeding coefficients calculated from pedigree relationships (Meuwissen and Luo, 1992). The genomic relationship matrix, **G**, was constructed as 0.95G* + 0.05**A**_22_; G* is defined according to VanRaden (2008) as follows:

[4] $${\text{G}}^{*}=\frac{\text{Z}{\text{Z}}^{\prime }}{\text{2}\sum_{i=1}^{M}p_{i}\left(1-p_{i}\right)},$$

where **Z** is a centered matrix of genotype at each locus (*aa* = 0, *Aa* = 1, and *AA* = 2), *M* is the number of SNP, and *p_i_* is the minor allele frequency at locus *i*.

To explore the genetic relationship between the 2 DCT traits (DCT-MAX and DCT-FT), bivariate models were fit for DCT-MAX and DCT-FT at each of the 4 time points. To estimate the genetic correlation between stages of production for each DCT trait, bivariate models were fit with each pairwise combination of time points (i.e., DCT-MAX at T1-Precalving and DCT-MAX at T2-Calving; DCT-FT at T1-Precalving and DCT-FT at T2-Calving, and so on), resulting in 6 bivariate models for DCT-MAX and 6 bivariate models for DCT-FT.

One of our objectives was to evaluate the genetic relationship between DCT and CHL. Therefore, we fit bivariate models for combinations of DCT and CHL traits, both within the same time point and between time points. Specifically, we fit bivariate models for DCT-MAX and SL-Severity at each of the 4 time points and for every pairwise combination of time points, such as DCT-MAX at T2-Calving and SL-Severity at T3-Early, and so on. This process was repeated for DCT-FT and SL-Severity, DCT-MAX and WL-Severity, and finally for DCT-FT and WL-Severity, resulting in an additional 40 bivariate models.

Bivariate models had the same parameters as the univariate models (Equation 1), and random effects were assumed to be normally distributed with a mean of zero and covariance structure of

[5] $$\mathrm{var}\left[\begin{array}{c} \text{a}\\ \text{e} \end{array}\right]=\left[\begin{array}{cc} {\text{G}}_{0}⊗\text{H} & 0\\ 0 & {\text{R}}_{0}⊗\text{I} \end{array}\right],$$

where **G**_0_ is the genetic covariance matrix between traits due to animal additive genetic effects, **R**_0_ is the residual covariance matrix between traits, ⊗ is the Kronecker product, **H** is the relationship matrix, and **I** is an identity matrix.

### Genomic Breeding Values and GWA Analyses

To address our objective of identifying QTL associated with DCT, we followed a single-step GWA study approach for each DCT trait (DCT-MAX and DCT-FT) at each of the 4 time points (Wang et al., 2012). The genetic background of CHL was beyond the scope of this study. First, single-step genomic best linear unbiased prediction was implemented in BLUPF90 (Misztal et al., 2018) to calculate estimated genomic breeding values for DCT-MAX and DCT-FT at each time point. Second, the genomic breeding values for DCT-MAX and DCT-FT were back-solved to estimate individual marker effects and *P*-values, using POSTGSF90 (Aguilar et al., 2014, 2019).

In each GWA analysis, genomic inflation was assessed by calculating the inflation factor of the test statistic (Amin et al., 2007). We adjusted for multiple testing with a Bonferroni correction that was considered appropriate for this study given the sample size, genotype density, correlation structure between markers, and the reported polygenic background of DCT. Significant SNP were defined using a statistical significance threshold of *P* ≤ 0.05, which was corrected for multiple testing to 7.67E-07 (*P* ≤ 0.05/number of tested markers). Suggestive SNP were defined using a genome-wide threshold equivalent to one false-positive result per genome scan (Lander and Kruglyak, 1995), the suggestive threshold was 1.53E-05 (*P* ≤ 1/number of tested markers).

A window-based GWA approach was used to further explore the association between genomic regions and DCT traits (DCT-MAX and DCT-FT). The window size was determined by linkage disequilibrium (**LD**) in our study population (Silva et al., 2020). The magnitude and decay of LD between SNP was evaluated using PLINK (Purcell et al., 2007). On average, LD was found to decay by 50% every 0.65 Mb; therefore, sliding windows of 0.65 Mb were used for window-based analyses. The proportion of genetic variance explained by each sliding window of 0.65 Mb was calculated using POSTGSF90, as described by Wang et al. (2014).

### QTL and Functional Analysis

Positional candidate genes were identified using the latest assembly of the *B. taurus* genome (ARS-UCD 1.2) downloaded from the National Center for Biotechnology Information database (https://www.ncbi.nlm.nih.gov/assembly/GCF_002263795.1/). In each GWA analysis, the closest gene to each significant or suggestive SNP was identified, up to a maximum of 0.2 Mb upstream or downstream from the marker. Additionally, genes were explored if they were contained or partially contained within genomic windows that explained >0.5% of the total genetic variance. The UniProt database (UniProt Consortium, 2021) was used for functional annotation of positional candidate genes. Enrichment analysis of the candidate genes from single-marker and window-based GWA analyses was conducted using the DAVID bioinformatic resource (Huang et al., 2009a, 2009b).

## RESULTS

### Population and Data Set Description

A total of 2,352 animals were enrolled in this study: 132 animals from herd A, 432 animals from herd B, 1,549 animals from herd C, and 239 animals from herd D. Details of the final number of animals with phenotype records at each assessment time point and the timing of each assessment relative to parturition are provided in Table 2. In some cases (n = 38), animals were enrolled before parturition but did not subsequently calve because they aborted, died, or were euthanized for health reasons. These animals were excluded from further analysis owing to the absence of a calving date, despite having phenotypes recorded at T1-Precalving. Additionally, to ensure environmental factors were broadly consistent at each time point, records were excluded from each time point if they fell outside of the planned sampling time frame (see ranges in Table 2, number of excluded records: T1-Precalving, n = 26; T2-Calving, n = 1; T3-Early, n = 8; T4-Late, n = 6). Most records that fell outside the planned sampling time frame were at T1-Precalving because animals were enrolled based on farm records of expected calving dates, which were occasionally inaccurate. A higher number of animals were lost to follow-up between T3-Early and T4-Late owing to a break in data collection due to COVID-19 restrictions. In sporadic instances, all 4 feet were not assessed for lesions at a time point owing to behavior of the animal in the foot-trimming crush that risked the safety of the animal or researchers. In the final data set, 99.5% (2,266/2,277), 99.2% (2,108/2,224), and 96.7% (1,868/1,931) of animals had lesion records from all 4 feet at T1-Precalving, T3-Early, and T4-Late, respectively. At T2-Calving, although 99.0% (2,164/2,185) of animals had lesion records from both hind feet, only 33.6% (734/2,185) had lesion records from all 4 feet, owing to the change in data collection procedure on herd C at this time point. Measurement of DCT at the 2 predetermined anatomical locations (DCT-MAX and DCT-FT) was not always possible with high precision and confidence because of the absence or ambiguity of necessary landmarks; in these cases, no measurement was recorded to ensure that the DCT phenotypes were as accurate as possible. The DCT-FT measurement was missing more frequently than the DCT-MAX measurement owing to difficulties in clearly identifying the point of the flexor tuberosity of the distal phalanx in the stored images.

**Table 2:** Details of data collection at each assessment time point including the timing of each assessment relative to calving date, the number of feet assessed, digital cushion images collected, and digital cushion thickness measurements from each animal^1^

Item	Parameter	Assessment time point^2^			
		T1-Precalving	T2-Calving	T3-Early	T4-Late
Timing of assessment relative to parturition (d)	Mean (SD)	−55.2 (18.9)	+5.4 (2.8)	+84.0 (13.6)	+200.0 (31.0)
	Range	−119 to −1	0 to 21	50 to 120	170 to 307
Total number of feet assessed from each animal	1 foot only	1	21	1	2
	2 feet only	4	1,427	2	22
	3 feet only	6	3	13	39
	All 4 feet	2,266	734	2,108	1,868
	Total	2,277	2,185	2,124	1,931
Digital cushion image collected		2,194	2,139	2,061	1,419
Measurement of DCT-MAX		2,091	2,066	1,995	1,380
Measurement of DCT-FT		1,059	1,157	1,020	670

1 DCT-FT = digital cushion thickness distal to the flexor tuberosity of the distal phalanx; DCT-MAX = maximum digital cushion thickness.

2 T1-Precalving = before parturition, T2-Calving = immediately after parturition, T3-Early = early lactation, T4-Late = late lactation.

Descriptive details of the phenotypes are provided in Table 3. Both DCT measurements (DCT-MAX and DCT-FT) followed a similar trend during the production cycle in both primiparous and multiparous animals: the thinnest DCT was recorded at T2-Calving and the thickest at T4-Late. At each time point, the Pearson correlation coefficient between DCT-MAX and DCT-FT ranged from 0.63 to 0.74. In primiparous animals, the prevalence of SH (all severity grades) was highest at T3-Early (392/582, 67.4%); in multiparous animals it was highest and similar at both T3-Early (830/1,542, 53.8%) and T4-Late (758/1,375, 55.1%). The prevalence of SU followed a similar pattern: the highest prevalence of SU (all severity grades) in primiparous animals was at T3-Early (22/582, 3.8%) and in multiparous animals it was highest and similar at T3-Early (107/1,542, 6.9%) and T4-Late (106/1,375, 7.7%). The prevalence of WL was highest at T4-Late for both primiparous (304/556, 54.7%) and multiparous animals (839/1,375, 61%).

**Table 3:** Mean (SD) of each trait at each time point: the maximum digital cushion thickness (DCT-MAX), the digital cushion thickness distal to the flexor tuberosity of the distal phalanx (DCT-FT), the mean sole lesion severity across all feet (SL-Severity), and the mean white line lesion severity across all feet (WL-Severity)^1^

Trait	Parity	T1-Precalving	T2-Calving	T3-Early	T4-Late
DCT-MAX, mm	Primiparous	5.98 (0.79)	5.92 (0.88)	6.26 (0.77)	7.01 (0.78)
	Multiparous	7.10 (0.93)	6.73 (0.86)	7.01 (0.90)	7.23 (0.91)
DCT-FT, mm	Primiparous	4.95 (0.70)	4.61 (0.77)	5.23 (0.83)	5.80 (0.80)
	Multiparous	5.93 (0.99)	5.43 (0.95)	5.80 (1.00)	6.04 (0.97)
SL-Severity (0–6)	Primiparous	0.12 (0.21)	0.18 (0.34)	0.48 (0.50)	0.24 (0.36)
	Multiparous	0.20 (0.39)	0.26 (0.55)	0.37 (0.49)	0.40 (0.57)
WL-Severity (0–3)	Primiparous	0.18 (0.28)	0.20 (0.32)	0.12 (0.22)	0.27 (0.33)
	Multiparous	0.12 (0.22)	0.17 (0.33)	0.18 (0.27)	0.29 (0.31)

1 T1-Precalving = before parturition, T2-Calving = immediately after parturition, T3-Early = early lactation, T4-Late = late lactation.

### Genetic Parameters

Four traits (DCT-MAX, DCT-FT, SL-Severity, and WL-Severity) were analyzed at 4 assessment time points with single-trait models, and estimates of variance components and heritability are provided in Table 4. Genetic correlation was estimated with bivariate models. The 2 DCT traits (DCT-MAX, DCT-FT) had strong genetic correlation with each other at each time point, with estimates ranging from 0.95 (±0.03) to 1.00 (±0.001). The genetic correlation between time points was between 0.92 (±0.04) and 1.00 (±0.001) for DCT-MAX, and between 0.88 (±0.26) and 1.00 (±0.01) for DCT-FT.

**Table 4:** Additive genetic variance $\left(\sigma_{a}^{2}\right),$ residual variance $\left(\sigma_{e}^{2}\right),$ and narrow-sense heritability (h^2^) estimates (SE) from single-trait analysis at each time point for the maximum digital cushion thickness (DCT-MAX), the digital cushion thickness distal to the flexor tuberosity of the distal phalanx (DCT-FT), the mean sole lesion severity across all feet (SL-Severity), and the mean white line lesion severity across all feet (WL-Severity)

Trait	Time point^1^	Number of animals	$\sigma_{a}^{2}$	$\sigma_{e}^{2}$	h^2^
DCT-MAX	T1-Precalving	2,091	0.18 (0.03)	0.61 (0.03)	0.23 (0.04)
	T2-Calving	2,066	0.21 (0.03)	0.52 (0.03)	0.29 (0.04)
	T3-Early	1,995	0.21 (0.03)	0.51 (0.03)	0.29 (0.04)
	T4-Late	1,380	0.32 (0.05)	0.40 (0.04)	0.44 (0.06)
DCT-FT	T1-Precalving	1,059	0.11 (0.05)	0.67 (0.05)	0.14 (0.06)
	T2-Calving	1,157	0.21 (0.05)	0.58 (0.05)	0.26 (0.06)
	T3-Early	1,020	0.12 (0.05)	0.68 (0.05)	0.15 (0.06)
	T4-Late	670	0.21 (0.08)	0.52 (0.07)	0.29 (0.10)
SL-Severity	T1-Precalving	2,277	0.018 (0.004)	0.09 (0.004)	0.16 (0.03)
	T2-Calving	2,185	0.028 (0.006)	0.21 (0.008)	0.12 (0.03)
	T3-Early	2,124	0.043 (0.009)	0.17 (0.008)	0.20 (0.04)
	T4-Late	1,931	0.038 (0.009)	0.20 (0.010)	0.16 (0.04)
WL-Severity	T1-Precalving	2,277	0.005 (0.002)	0.05 (0.002)	0.09 (0.03)
	T2-Calving	2,185	0.007 (0.003)	0.10 (0.004)	0.07 (0.03)
	T3-Early	2,124	0.007 (0.002)	0.05 (0.002)	0.11 (0.03)
	T4-Late	1,931	0.013 (0.001)	0.09 (0.004)	0.13 (0.04)

1 T1-Precalving = before parturition, T2-Calving = immediately after parturition, T3-Early = early lactation, T4-Late = late lactation.

The genetic correlations between DCT traits (DCT-MAX, DCT-FT) and CHL traits (SL-Severity, WL-Severity), both within each time point and between time points, are provided in Table 5, Table 6. The genetic correlation between DCT-MAX and SL-Severity was generally negative; the 95% confidence interval of this estimate did not include zero on 5 occasions: between DCT-MAX at T2-Calving and SL-Severity at T3-Early and T4-Late (−0.33 and −0.37, respectively), between DCT-MAX at T3-Early and SL-Severity at T3-Early and T4-Late (−0.33 and −0.38, respectively), and between DCT-MAX and SL-Severity at T4-Late (−0.35). The genetic correlation between DCT-FT and SL-Severity followed a similar pattern, but the 95% confidence interval of this estimate did not include zero on only 2 occasions: between DCT-FT at T2-Calving and SL-Severity at T3-Early and T4-Late (−0.44 and −0.30, respectively). The genetic correlation between DCT traits and WL-Severity was effectively zero (95% confidence interval of estimate included zero) on all occasions except between DCT-MAX at T3-Early and WL-Severity at T4-Late (0.29).

**Table 5:** The additive genetic correlation (SE) between the maximum digital cushion thickness (DCT-MAX) or the digital cushion thickness distal to the flexor tuberosity of the distal phalanx (DCT-FT) and mean severity of sole lesions across all feet (SL-Severity)^1^

Trait	Time point^2^	SL-Severity			
		T1-Precalving	T2-Calving	T3-Early	T4-Late
DCT-MAX	T1-Precalving	−0.12 (0.12)	−0.23 (0.12)	−0.17 (0.11)	−0.18 (0.12)
	T2-Calving		−0.12 (0.12)	−0.33 (0.10)*	−0.37 (0.11)*
	T3-Early			−0.33 (0.10)*	−0.38 (0.11)*
	T4-Late				−0.35 (0.11)*
DCT-FT	T1-Precalving	0.17 (0.22)	0.06 (0.22)	−0.11 (0.22)	−0.09 (0.23)
	T2-Calving		−0.11 (0.15)	−0.44 (0.12)*	−0.30 (0.14)*
	T3-Early			−0.47 (0.46)	−0.07 (0.23)
	T4-Late				−0.37 (0.42)

1 Values on the diagonal refer to the genetic correlation between traits that were both recorded at the same time point, and those above the diagonal refer to the genetic correlation between traits that were recorded at different time points.

2 T1-Precalving = before parturition, T2-Calving = immediately after parturition, T3-Early = early lactation, T4-Late = late lactation.

* The 95% confidence interval does not include zero.

**Table 6:** The additive genetic correlation (SE) between the maximum digital cushion thickness (DCT-MAX) or the digital cushion thickness distal to the flexor tuberosity of the distal phalanx (DCT-FT) and mean severity of white line lesions across all feet (WL-Severity)^1^

Trait	Time point^2^	WL-Severity			
		T1-Precalving	T2-Calving	T3-Early	T4-Late
DCT-MAX	T1-Precalving	0.32 (0.16)	0.04 (0.05)	0.23 (0.16)	−0.08 (0.14)
	T2-Calving		0.04 (0.17)	0.18 (0.13)	−0.04 (0.13)
	T3-Early			0.13 (0.14)	0.29 (0.14)*
	T4-Late				0.21 (0.14)
DCT-FT	T1-Precalving	0.42 (0.47)	0.29 (0.55)	0.44 (0.54)	0.26 (0.28)
	T2-Calving		0.41 (0.27)	0.34 (0.18)	−0.03 (0.18)
	T3-Early			0.45 (0.32)	0.46 (0.30)
	T4-Late				0.40 (0.38)

1 Values on the diagonal refer to the genetic correlation between traits that were both recorded at the same time point, and those above the diagonal refer to the genetic correlation between traits which were recorded at different time points

2 T1-Precalving = before parturition, T2-Calving = immediately after parturition, T3-Early = early lactation, T4-Late = late lactation.

* The 95% confidence interval does not include zero.

### QTL and Functional Analysis

**Figure 1:**
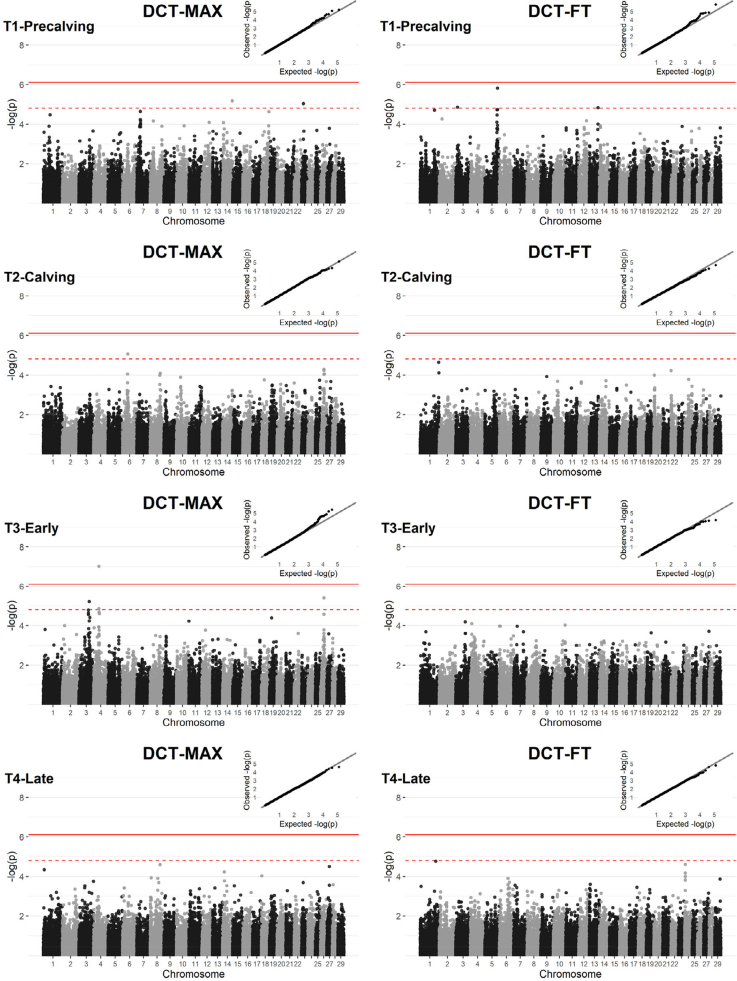
Manhattan plots and quantile-quantile plots of the maximum digital cushion thickness (DCT-MAX) and the digital cushion thickness distal to the flexor tuberosity of the distal phalanx (DCT-FT) at each time point (T1–T4); −log_10_*P*-value of marker effects against marker position on the chromosome. T1-Precalving = before parturition, T2-Calving = immediately after parturition, T3-Early = early lactation, T4-Late = late lactation. The solid line represents the genome-wide significance threshold (*P* ≤ 7.67E-07, 0.05/number of tested markers), and the dashed line represents the suggestive threshold (*P* ≤ 1.53E-05, 1/number of tested markers).

GWA analysis was performed for each DCT trait (DCT-MAX, DCT-FT) at each time point. The genomic inflation factor ranged from 0.94 to 1.00 in the 8 single-marker GWA analyses. Single-marker analyses revealed a polygenic background to both DCT-MAX and DCT-FT at each time point (Figure 1). From all GWA analyses, only one significant SNP was identified: for DCT-MAX at T3-Early on *B. taurus* autosome (**BTA**)-4. Suggestive SNP were identified on BTA-3, BTA-5, BTA-6, BTA-13, BTA-14, BTA-23, and BTA-26 (Table 7). Positional candidate genes that were located closest to each suggestive or significant SNP, and within 0.2 Mb upstream or downstream of the marker, are presented in Table 7.

**Table 7:** Markers with a significant (*P* ≤ 7.67E-07, 0.05/number of tested markers) or suggestive effect (*P* ≤ 1.53E-05, 1/number of tested markers) on maximum digital cushion thickness (DCT-MAX) or digital cushion thickness distal to the flexor tuberosity of the distal phalanx (DCT-FT) at each time point, with respective chromosome (BTA), position (bp), minor allele frequency (MAF), *P*-value of marker effect, and name and location of the closest gene up to a maximum of 0.2 Mb upstream or downstream from the marker

Trait	Time point^1^	BTA	Position (bp)	MAF	*P*-value	Gene	Gene location
DCT-MAX	T1-Precalving	14	81,367,974	0.25	6.58E-06	*DEPTOR*^2^	81,286,837–81,402,413
		23	16,504,326	0.33	8.96E-06	*BICRAL*^2^	16,471,820–16,506,078
	T2-Calving	6	42,273,485	0.19	8.43E-06	*GBA3*^2^	42,263,666–42,395,508
	T3-Early	3	84,753,032	0.27	6.05E-06	*NFIA*	84,203,793–84,620,790
		4	44,839,097	0.32	9.56E-08	*RELN*^2^	44,652,801–45,211,015
		26	28,997,874	0.48	3.96E-06	*—*	—
DCT-FT	T1-Precalving	3	15,443,604	0.08	1.37E-05	*TRIM46*	15,433,548–15,443,291
		5	104,055,417	0.49	1.51E-06	*TNFRSF1A*	104,024,027–104,036,846
		13	71,799,248	0.22	1.48E-05	*—*	—

1 T1-Precalving = before parturition, T2-Calving = immediately after parturition, T3-Early = early lactation, T4-Late = late lactation.

2 Marker was located inside candidate gene.

**Figure 2:**
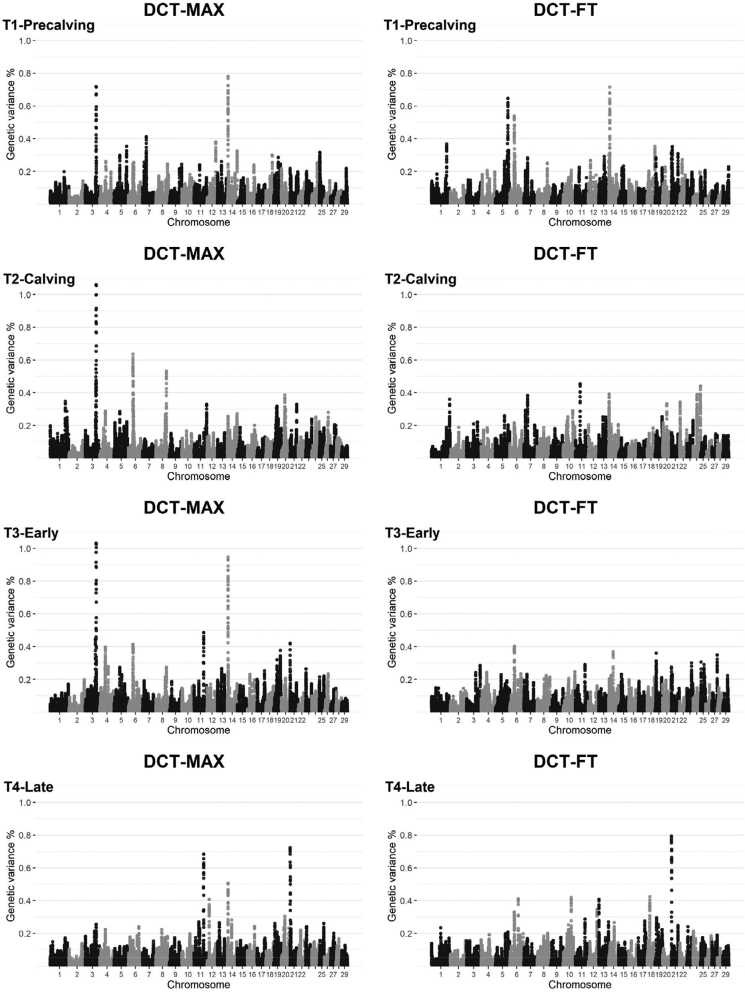
Manhattan plots of the proportion of the total additive genetic variance explained by sliding 0.65-Mb windows for the maximum digital cushion thickness (DCT-MAX) and the digital cushion thickness distal to the flexor tuberosity of the distal phalanx (DCT-FT) at each time point (T1–T4). T1-Precalving = before parturition, T2-Calving = immediately after parturition, T3-Early = early lactation, T4-Late = late lactation.

Window-based GWA analyses showed a similarly complex genetic background to DCT-MAX and DCT-FT (Figure 2). The same genomic window on BTA-3 (comprising 31 SNP) explained more than 1% of the total genetic variance for DCT-MAX at T2-Calving and T3-Early, and a neighboring window (comprising 29 SNP) explained 0.72% of total genetic variance for DCT-MAX at T1-Precalving. Other genomic windows that explained more than 0.5% of the total genetic variance were identified on BTA-5, BTA-6, BTA-8, BTA-11, BTA-14, and BTA-21 (Table 8). All genes that were contained or partially contained within these genomic windows are presented in Table 8.

**Table 8:** Genomic regions that explained more than 0.5% of the total additive genetic variance for the maximum digital cushion thickness (DCT-MAX) and the digital cushion thickness distal to the flexor tuberosity of the distal phalanx (DCT-FT) at each time point, with respective chromosome (BTA), window position (bp), proportion of total genetic variance explained, and the name of gene(s) contained (or partially contained) within these windows

Trait	Stage^1^	BTA	Window position (bp)	Variance (%)	Gene
DCT-MAX	T1-Precalving	3	90,742,849–91,356,392	0.72	*BSND*, *TMEM61*
		14	5,880,036–6,526,644	0.78	*KHDRBS3*
	T2-Calving	3	90,725,628–91,356,392	1.06	*BSND*, *TMEM61*
		6	37,690,172–38,332,952	0.64	—
		8	88,134,972–88,775,323	0.53	*GADD45G*, *SEMA4D*
	T3-Early	3	90,725,628–91,356,392	1.03	*BSND*, *TMEM61*
		14	5,880,036–6,526,644	0.95	*KHDRBS3*
	T4-Late	11	78,811,939–79,407,746	0.68	*LAPTM4A*, *TTC32*, *OSR1*
		14	5,998,335–6,619,386	0.51	*KHDRBS3*
		21	2,416,354–3,040,671	0.72	—
DCT-FT	T1-Precalving	5	103,798,423–104,430,699	0.65	*LPAR5*, *CHD4*, *NOP2*, *IFFO1*, *GAPDH*, *NCAPD2*, *MRPL51*, *VAMP1*, *TAPBPL*, *CD27*, *LTBR*, *SCNN1A*, *TNFRSF1A*, *CD9*, *VWF*
		6	37,796,921–38,426,291	0.54	—
		14	8,954,477–9,597,429	0.72	KCNQ3, EFR3A
	T4-Late	21	2,416,354–3,040,671	0.79	—

1 T1-Precalving = before parturition, T2-Calving = immediately after parturition, T3-Early = early lactation, T4-Late = late lactation.

The set of positional candidate genes, identified from single-marker and window-based GWA analyses, were enriched in biological processes of 5 Gene Ontology (**GO**) terms: positive regulation of I-kappaB kinase/NF-kappaB signaling (GO: 0043123), positive regulation of CREB transcription factor activity (GO: 0032793), cellular response to mechanical stimulus (GO: 0071260), cell adhesions (GO: 0007155), and positive regulation of phosphatidylinositol 3-kinase signaling (GO: 0014068).

## DISCUSSION

### Key Results and Interpretation: Genetic Parameters

We have corroborated the results of previous studies that demonstrated that DCT, as measured using ultrasonography, is a heritable trait in Holstein cows (Oikonomou et al., 2014; Sánchez-Molano et al., 2019; Stambuk et al., 2020a,b). As such, scope could exist for increasing the average thickness of the digital cushion in a population through selective breeding, which may translate to a reduced incidence of CHL (Machado et al., 2011; Toholj et al., 2014; Newsome et al., 2017a; Stambuk et al., 2018; Griffiths et al., 2020).

The heritability estimates in our study tended to be higher for the maximum DCT measurement (DCT-MAX, heritability: 0.23–0.44) compared with the DCT measurement taken distal to the flexor tuberosity of distal phalanx (DCT-FT, heritability: 0.14–0.29). The DCT-FT measurement is arguably more clinically relevant than DCT-MAX for sole lesion development, as this corresponds to the predilection site for these lesions and DCT-FT has been shown to correlate with CHL risk phenotypically (Machado et al., 2011; Toholj et al., 2014; Newsome et al., 2017a; Griffiths et al., 2020). We also observed a trend whereby the heritability of the DCT was dependent on the stage of lactation, with the heritability of DCT (both DCT-MAX and DCT-FT) being lowest at T1-Precalving and highest at T4-Late. These differences are partially a reflection of changes in environmental variance; however, the additive genetic variance was highest at the T4-Late assessment. This trend is difficult to explain biologically, and previous studies have not reported genetic parameters for DCT from different lactation stages (Oikonomou et al., 2014; Sánchez-Molano et al., 2019; Stambuk et al., 2020a,b); therefore, we do not know if this observation is consistent in other populations. Overall, the heritability estimates of DCT in our study (both DCT-MAX and DCT-FT) were broadly comparable to previous studies of Holsteins in which estimates ranged from 0.23 to 0.33 (Oikonomou et al., 2014; Sánchez-Molano et al., 2019; Stambuk et al., 2020b).

A key objective of our study was to estimate the genetic correlation between DCT and CHL. The genetic correlation between DCT traits and WL-Severity was not statistically different from zero, except between DCT-MAX at T3-Early and WL-Severity at T4-Late when it was positive (0.29 ± 0.14). This single positive genetic correlation should be interpreted cautiously owing to the large standard errors of this estimate, and because this was the only nonzero genetic correlation between these traits. If a positive genetic correlation truly exists between DCT and WL, it may reflect previous observations of a potentially positive genetic correlation between body condition and DCT (Oikonomou et al., 2014), a positive genetic correlation between body condition and body weight (Berry et al., 2003), and a positive phenotypic association between body weight and WL (Schöpke et al., 2013; Pérez-Cabal and Charfeddine, 2016). This explanation is convoluted, in part because the direct relationship between body condition and WL has not been established. Therefore, more research would be beneficial to assess the relationship between body condition and WL, as well as the genetic and phenotypic relationships between DCT and WL.

Our results indicate that DCT traits were negatively correlated with SL-Severity at several time points, although the magnitude of the genetic correlation in these instances was relatively small. A negative genetic correlation between DCT and CHL incidence was reported by Oikonomou et al. (2014), and our results imply that this relationship may have been due to a negative genetic correlation between DCT and sole lesions (SH and SU), rather than between DCT and WL. Correlated traits can be incorporated into a composite selection index to increase the accuracy of selection and improve genetic progress (Boettcher et al., 1998; Banos et al., 2006); therefore, including DCT in selection indexes for claw health could be beneficial. However, DCT is more challenging to record than foot lesions, so this is unlikely to be practical unless DCT was recorded in an intensively monitored reference population (Pryce et al., 2012; Calus et al., 2013; Coffey, 2020).

The strength of the genetic correlation between DCT traits and SL-Severity that we observed was generally weak, with large standard errors, and in many cases the 95% confidence interval included zero. Unbiased estimates of genetic correlation are effectively impossible to obtain (Lynch and Walsh, 1998), therefore care should be taken not to over-interpret the magnitude of these estimates or extrapolate them across populations. However, we consider the occasions in which the 95% confidence interval of the correlation estimate did not include zero to be the most persuasive evidence for a truly negative relationship between the additive genetic variance of both traits; these specific results are interesting in the context of the proposed pathogenesis of sole lesion development.

We observed a weak negative genetic correlation between the DCT immediately after calving and the severity of sole lesions later in lactation. The suspensory apparatus of the distal phalanx has been shown to be weaker around parturition, which may result in compression of the underlying soft tissues, including the digital cushion and corium (Tarlton et al., 2002; Knott et al., 2007). Compression of the corium is thought to be detrimental to claw horn production and to initiate the development of SH or SU (Lischer and Ossent, 2002; Lischer et al., 2002). The weak negative genetic correlation between DCT immediately after calving and future sole lesions lends support to this hypothesis from a genetic perspective. However, correlation is a bidirectional relationship, and therefore, an alternative explanation also exists. The development of CHL has been hypothesized to use fatty acids from the digital cushion as inflammatory mediators, thereby reducing the adipose tissue in the digital cushion, causing a reduction in thickness and a presumed impairment of its functionality (Ossent and Lischer, 1998; Lischer et al., 2002; Räber et al., 2006). Development of CHL has also been shown to increase the risk of future CHL development (Hirst et al., 2002; Oikonomou et al., 2013); therefore, the weak genetic correlation we observed between DCT immediately after calving and sole lesions later in lactation could be due to the occurrence of previous sole lesions. Furthermore, the genetic background to these historic lesions could affect future DCT and sole lesion risk.

Periparturient compression of the digital cushion is an intuitive explanation for our observation that DCT was thinnest immediately after calving, a finding replicated elsewhere (Newsome et al., 2017b; Bach et al., 2021). Our results indicate that DCT was strongly genetically correlated between stages of lactation, and the additive genetic variance estimates were similar at T2-Calving to other time points. Therefore, it would appear that no major genetic component explains why the DCT is thinnest immediately after calving beyond the genetic background to DCT that exists generally. We conclude from these results that the extent of periparturient laxity in the suspensory apparatus of the distal phalanx and the associated compression of soft tissues are more likely to be determined by environmental factors than genetic.

We recorded the DCT to be thinner at T3-Early than at either T1-Precalving or T4-Late, in agreement with previous research (Bicalho et al., 2009; Newsome et al., 2017b; Griffiths et al., 2020). High-yielding dairy cows mobilize extreme quantities of fat in early lactation (McNamara, 1991; Drackley et al., 2006) and the digital cushion is primarily composed of adipose tissue (Räber et al., 2004, 2006). Lipolysis during early lactation has been suggested to deplete the adipose tissue of the digital cushion and lead to the observed reduction in DCT at this time, which in turn, is linked to an increased risk of CHL development (Bicalho et al., 2009). We observed a weak negative genetic correlation between DCT at T3-Early and T4-Late, and the severity of sole lesions at both T3-Early lactation and T4-Late time points. These observations suggest that the genetic tendency to have a thin DCT in early and late lactation is correlated with the genetic predisposition to develop more severe sole lesions at these times. However, the phenotypic relationships between fat mobilization, DCT, and CHL development are complicated, and reduced subcutaneous backfat is also associated with increased CHL risk independent of DCT (Newsome et al., 2017a). Therefore, it would be interesting for future studies to consider the genetic relationships between subcutaneous fat, DCT, and CHL development.

### Key Results and Interpretation: QTL Mapping

Characterization of the genetic background of DCT revealed a complex trait, in agreement with previous research (Sánchez-Molano et al., 2019; Stambuk et al., 2020a,b). The marker with the strongest evidence of an effect on DCT was observed on BTA-4 for DCT-MAX at T3-Early. This SNP is situated within the *RELN* gene. In cattle, *RELN* is primarily expressed in central nervous tissue and is involved in neuron development (Fang et al., 2020; UniProt Consortium, 2021). Although a link between the central nervous system and the digital cushion is not immediately intuitive, the *RELN* gene was involved in 3 out of the 5 enriched biological pathways from the analysis of all candidate genes. These biological pathways were CREB transcription factor activity, cell adhesion, and phosphatidylinositol 3-kinase signaling. In dairy cattle, CREB is associated with periparturient lipid metabolism and is considered to be key regulator of adipogenesis (McNamara et al., 1992). Phosphatidylinositol 3-kinase, an enzyme involved in insulin signaling, mediates glucose and lipid metabolism (Shepherd et al., 1998). Therefore, CREB transcription factor activity and phosphatidylinositol 3-kinase signaling pathways could plausibly relate to the thickness of the digital cushion and may underlie the observed association between DCT and the *RELN* gene, although this possibility requires further investigation.

Many of the candidate genes we identified have roles in inflammation. In humans, *RELN* has been associated with bone development due to inflammation (Garshasbi et al., 2020), as has another candidate gene, *SEMA4D*, which was identified for DCT-MAX at T2-Calving (Lontos et al., 2018). Localized inflammation in the bovine hoof has been linked with the development of bone growth on the distal phalanx (Lischer et al., 2002; Newsome et al., 2016), and genes relating to inflammation and bone growth have previously been associated with DCT in dairy cattle (Stambuk et al., 2020a,b). One of the enriched biological processes was I-κB kinase/NF-κB signaling, a complex pathway that is instrumental in a wide range of inflammatory responses (Ghosh and Hayden, 2008; Liu et al., 2017). This pathway is recognized to have an important role in inflammatory osteolysis in humans (Boyce et al., 2010; Abu-Amer, 2013) and in inflammation in diseases linked to lipid metabolism (Barma et al., 2009; Baker et al., 2011). In cattle, ketone bodies, which are produced from the metabolism of fatty acids, are reported to activate the NF-κB pathway in bovine hepatocytes (Shi et al., 2014). It is possible that genetic regulation of inflammation, particularly if it is also associated with bone changes or lipid metabolism, could directly or indirectly influence the digital cushion.

The NF-κB pathway has been shown to be activated by LPS, and this pathway has been linked to subacute ruminal acidosis and clinical mastitis in dairy cows (Fan et al., 2016; Khan et al., 2020). An in vitro study demonstrated LPS caused inflammation of the dermal cells in the bovine hoof (Tian et al., 2019), and systemic administration of LPS in vivo has been reported to induce histological changes in the laminae (Boosman et al., 1991). Inflammation of the laminae is hypothesized to cause laxity in the suspensory apparatus of the distal phalanx (Ossent and Lischer, 1998), which would result in compression of the digital cushion.

Although candidate genes or biological pathways with roles in inflammation may affect the digital cushion, the DCT has been reported to increase when CHL were present owing to inflammation in the corium (Ossent and Lischer, 1998; Newsome et al., 2017b). Therefore, as the DCT measurement in our study included both the digital cushion and the corium, genes associated with inflammation may be affecting DCT because of inflammation in the corium, rather than directly affecting the thickness of the digital cushion.

The genomic region that explained the greatest proportion of the total genetic variance for DCT at any time point was on BTA-3 (90.73–91.36 Mb). This region explained 1.06% and 1.03% of the total genetic variation of DCT-MAX at T2-Calving and T3-Early, respectively, and contained 2 candidate genes, *BSND* and *TMEM61*. The *BSND* gene is associated with chloride transport and *TMEM61* is unannotated (UniProt Consortium, 2021); therefore, it is not clear how these genes may relate to the digital cushion. However, QTL in this window have previously been associated with fat percentage and mineral content of milk in dairy cattle (Buitenhuis et al., 2015; van den Berg et al., 2020) and with luteal activity in early lactation (Tenghe et al., 2016). Therefore, this part of the genome may be worth further investigation in dairy cattle.

Other results of interest from GWA analyses include the genomic region on BTA-14 (5.88–6.53 Mb), which explained 0.78% and 0.95% of the total genetic variance for DCT-MAX at T1-Precalving and T3-Early, respectively. This window was also adjacent to a region (6.00–6.62 Mb) that explained 0.51% of the total genetic variance for DCT-MAX at T4-Late. The candidate gene in these windows was *KHDRBS3.* This gene has been associated with average daily gain in cattle (Seabury et al., 2017), and it is in LD with a neighboring gene associated with intramuscular fat deposition (Barendse et al., 2004; Bovine HapMap Consortium et al., 2009). The digital cushion is primarily composed of adipose tissue, and *KHDRBS3* is expressed in both adipose and muscle tissue in cattle (Räber et al., 2006; Fang et al., 2020). Therefore, an association between *KHDRBS3* and DCT is biologically plausible. The *KHDRBS3* gene was also associated with SU development in a GWA study of CHL using the same data set (B. Li, SRUC, Edinburgh, UK, personal communication), so *KHDRBS3* may contribute to the genetic correlation between DCT and sole lesions.

The other candidate genomic region on BTA-14 (8.95–9.60 Mb), which explained 0.72% of the total genetic variation for DCT-FT at T1-Precalving, was also associated with SU development in GWA analysis of CHL using this data set (B. Li, SRUC, Edinburgh, UK, personal communication). Candidate genes in this window were *KCNQ3* and *EFR3A.* The *KCNQ3* gene has previously been associated with milk fat percentage and milk yield in Holsteins (Kolbehdari et al., 2008; Jiang et al., 2019), while *EFR3A* has previously been associated with subclinical ketosis, milk fat percentage, and milk fatty acid composition in Holsteins (Li et al., 2014; Jiang et al., 2019; Soares et al., 2021). The lipid composition of the digital cushion changes with age (Räber et al., 2006) and is correlated with body condition (Hiss-Pesch et al., 2019; Newsome et al., 2021). Although it is not clear how the composition of the digital cushion affects its physical properties, it is plausible that the same genes that affect the fat and fatty acid content of milk could similarly influence the digital cushion. Additionally, milk yield, body condition, and subclinical ketosis have all been linked to CHL development (Amory et al., 2008; Green et al., 2014; Sepúlveda-Varas et al., 2018), so *KCNQ3* and *EFR3A* may also contribute to the genetic correlation between DCT and sole lesions.

Two of the candidate genes highlighted for DCT have previously been associated with conformation traits in cattle. The *OSR1* gene on BTA-11 (identified for DCT-MAX at T4-Late) has been linked to multiple conformation traits including feet and leg conformation, rear leg placement, and rump width (Cole et al., 2011). The *VWF* gene on BTA-5 (identified for DCT-FT at T1-Precalving) has previously been associated with foot angle (Kolbehdari et al., 2008). The volume of the digital cushion has been shown to increase when growing calves are exercised on rough terrain (Gard et al., 2015), which implies the size of the digital cushion is affected by external forces. Therefore, genes that affect limb conformation could conceivably influence DCT.

We did not identify any QTL or candidate genes that were highlighted by previous GWA studies of DCT (Stambuk et al., 2020a,b). However, one candidate gene was *TRIM46* on BTA-3 (identified for DCT-FT at T1-Precalving), and one of the candidate genes for DCT identified by Stambuk et al. (2020b) was *TRIM55*. Although *TRIM46* and *TRIM55* are in different families (Short and Cox, 2006; Ozato et al., 2008), TRIM proteins are associated with immune responses (Yang and Xia, 2021), and therefore, a potential link exists between the genetic control of the immune system and DCT that would benefit from future research. Additionally, we highlighted QTL on BTA-3 (90.73–91.36 Mb) and BTA-14 (81.37 Mb), which are relatively close to QTL reported by Stambuk et al. (2020a) on BTA-3 (95.85–95.93 Mb) and BTA-14 (80.04–80.66 Mb); these genomic regions may also be worth further exploration.

Overall, in the context of the limited previous research in this area, our results replicate some of the reported findings as well as providing additional data. Given the number of markers tested in 8 GWA analyses, only a small number of markers were associated with significant or suggestive effects on DCT. Our results did not corroborate any of the specific QTL reported in 2 previous GWA studies of DCT by Stambuk et al. (2020a,b). Although the biological grouping and function of highlighted genes in our results were similar to those reported by Stambuk et al. (2020a,b), speculation about candidate genes relies heavily on the existing understanding of the biology of the trait in question; therefore, this agreement in terms of gene function would be expected.

### Study Strengths and Limitations

We have estimated the genetic parameters and characterized the genetic background of DCT using the largest data set currently available. In addition to the size of the study population, the prospective cohort study design and accuracy of phenotype recording are further strengths of this study; however, we acknowledge some important limitations.

One of the limitations of this study population was the small number of farms included, which could reduce estimates of environmental variance and inflate the estimated heritability. This limitation also affects previous research, so we have insufficient context for speculating about how much it affects our results. It is also important to note that almost two-thirds of the study population were from a single herd; replication of results in a wider and more diverse population would strengthen interpretation of our findings.

A relatively large proportion of animals had missing data for the DCT-FT measurement due to the absence or ambiguity of anatomical landmarks in the stored ultrasound images; we did not record DCT-FT from these images to maintain a high accuracy of this phenotype. We designed our study to collect and store images that were retrospectively blinded and measured. If we had measured images at the same time as data collection, we could have reduced the number of missing measurements; however, subconscious biases can influence this process unless measurements are made with blinding to factors such as stage of lactation, body condition, and presence of lesions (Griffiths et al., 2020). We applied stringent criteria to all stored images to ensure all DCT measurements were consistent and accurate, but this approach resulted in more missing DCT-FT measurements and reduced study power for this trait.

It has recently been shown, albeit in a small cohort of animals, that ultrasound measurements of DCT in weight-bearing and non-weight-bearing feet are only weakly correlated (Bach et al., 2021); therefore, the key assumption that our DCT measurements translate to DCT during standing and walking is potentially undermined. Wider concerns exist about the interpretation of ultrasound measurements of the digital cushion. A recent study quantified the volume of the digital cushion in cadavers using magnetic resonance imaging and found the volume of the digital cushion in the lateral claws of hindfeet to range from 0 to 30 mL, with the middle digital cushion pad often completely absent (Wilson et al., 2021). Authors of this study considered ultrasonographic DCT measurements to therefore relate exclusively to the corium in many cases, particularly when targeting the middle fat pad (i.e., DCT-FT) (Wilson et al., 2021). Previous estimates report corium thickness to be no more than approximately 3 to 4 mm on ultrasound images (Toholj et al., 2014; Newsome et al., 2017b). In our study, a DCT-FT measurement of 4 mm corresponded to the 5th percentile, implying that the DCT-FT measurements are unlikely to only represent the corium in 95% of cases. We would consider it more likely that these measurements also include connective tissue, which is reported to replace adipose tissue in the digital cushion (Ossent and Lischer, 1998; Lischer et al., 2002). This explanation would be more consistent with the negative correlation reported between the thickness and echotexture of the digital cushion (Bicalho et al., 2009) because the corium is anechoic (Kofler et al., 1999). Regardless, the relationship of the corium with CHL development is not fully understood. A thin corium has been associated with future CHL development (Toholj et al., 2014), whereas a thickened corium is associated with the presence of a concurrent CHL (Lischer et al., 2002; Newsome et al., 2017b). Interestingly, the ultrasonographic thickness of the corium has also been shown to correlate to subcutaneous fat thickness (Newsome et al., 2017b). This finding presents a further complication to the hypothesized pathogenesis of CHL, which appears to include at minimum the digital cushion, corium, and subcutaneous fat, as well as to the relationships between these factors.

In conclusion, it is reasonable to question whether DCT measured using ultrasonography is the most important property of the digital cushion in terms of force dissipation and CHL development. Previous studies have described the composition of the digital cushion and its relationship with body condition and foot lesions (Räber et al., 2006; Hiss-Pesch et al., 2019; İzci et al., 2019; Newsome et al., 2021), but the results are still equivocal or preliminary in terms of the implications for CHL development. Limitations exist in defining the ability of the digital cushion to effectively dissipate forces in the foot by its thickness, and key questions remain unanswered about how to infer the functionality of the digital cushion from either physical dimensions or its composition. It is also fair to say that the measurement of the digital cushion using ultrasound is likely to be an example of an observational bias, where its importance may have been overestimated owing to the relative ease of measurement. Further studies should attempt to use different approaches to assess the functionality of the digital cushion; unfortunately, no such techniques have yet been described that could be employed in the type of longitudinal study required to clarify the role of the digital cushion in the pathogenesis of CHL.

### Generalizability

Caution is required to generalize the genetic parameters and QTL reported in this study, particularly given the polygenic nature of DCT and the small number of herds. Our results are from a population of Holstein cows in 4 dairy herds that were all commercially run with operating practices common to many UK dairy farms, but they could not be considered representative of the full spectrum of dairy farms. Within these 4 herds, 3 were operating similar and relatively intensive systems of zero grazing and 3 times a day milking. The overall period prevalence of lame cows (Mahendran et al., 2017), based on repeated mobility scores throughout this project, ranged from 18.5% to 33.3% across the 4 herds; the mean point prevalence of lameness from all time points ranged from 6% to 11.8% across the 4 herds (data not shown). Recent cross-sectional studies in the United Kingdom reported that herd lameness prevalence ranged from 6% to 65%; this report suggests that the 4 herds in our study had a lower prevalence of lameness compared with many dairy herds in the United Kingdom (Griffiths et al., 2018; Randall et al., 2019).

## CONCLUSIONS

The results from this prospective cohort study indicate that DCT is a heritable trait that has a weak negative genetic correlation with the severity of sole lesions, but not with WL. The strength of the genetic correlation between DCT and sole lesions depends on the stage of lactation at which both the digital cushion and sole lesions are assessed. Digital cushion thickness is a polygenic trait, and few QTL were associated with observable effects. Candidate genes identified for DCT are related to inflammation, fat metabolism, and bone development. Most notably, the *KHDRBS3* gene may contribute to the susceptibility of cows to SU in addition to the thickness of the digital cushion. Further work is needed to investigate these candidate genes and establish the precise role of the digital cushion in the pathogenesis of sole lesions.
